# Machine Learning Prediction Model for Acute Renal Failure After Acute Aortic Syndrome Surgery

**DOI:** 10.3389/fmed.2021.728521

**Published:** 2022-01-17

**Authors:** Jinzhang Li, Ming Gong, Yashutosh Joshi, Lizhong Sun, Lianjun Huang, Ruixin Fan, Tianxiang Gu, Zonggang Zhang, Chengwei Zou, Guowei Zhang, Ximing Qian, Chenhui Qiao, Yu Chen, Wenjian Jiang, Hongjia Zhang

**Affiliations:** ^1^Department of Physiology and Pathophysiology, School of Basic Medical Sciences, Capital Medical University, Beijing, China; ^2^Department of Cardiac Surgery, Beijing Anzhen Hospital, Capital Medical University, Beijing, China; ^3^Beijing Lab for Cardiovascular Precision Medicine, Beijing, China; ^4^Department of Cardiothoracic Surgery, St Vincent's Hospital, Sydney, NSW, Australia; ^5^Department of Interference Diagnosis and Treatment, Beijing Anzhen Hospital, Capital Medical University, Beijing, China; ^6^Department of Cardiovascular Surgery, Guangdong Provincial People's Hospital, Guangzhou, China; ^7^Department of Cardiac Surgery, First Affiliated Hospital, China Medical University, Shenyang, China; ^8^Department of Cardiac Surgery, People's Hospital of Xinjiang Uygur Autonomous Region, Urumqi, China; ^9^Department of Cardiovascular Surgery, Shandong Provincial Hospital Affiliated With Shandong First Medical University, Jinan, China; ^10^Department of Cardiovascular Surgery, The First Affiliated Hospital of Harbin Medical University, Harbin, China; ^11^Department of Cardiac Surgery, School of Medicine, Sir Run Run Shaw Hospital, Zhejiang University, Hangzhou, China; ^12^Department of Cardiovascular Surgery, The First Affiliated Hospital of Zhengzhou University, Zhengzhou, China; ^13^Department of Cardiac Surgery, Peking University People's Hospital, Beijing, China

**Keywords:** machine learning, acute renal failure, acute aortic syndrome, prediction model, eXtreme Gradient Boosting

## Abstract

**Background:**

Acute renal failure (ARF) is the most common major complication following cardiac surgery for acute aortic syndrome (AAS) and worsens the postoperative prognosis. Our aim was to establish a machine learning prediction model for ARF occurrence in AAS patients.

**Methods:**

We included AAS patient data from nine medical centers (*n* = 1,637) and analyzed the incidence of ARF and the risk factors for postoperative ARF. We used data from six medical centers to compare the performance of four machine learning models and performed internal validation to identify AAS patients who developed postoperative ARF. The area under the curve (AUC) of the receiver operating characteristic (ROC) curve was used to compare the performance of the predictive models. We compared the performance of the optimal machine learning prediction model with that of traditional prediction models. Data from three medical centers were used for external validation.

**Results:**

The eXtreme Gradient Boosting (XGBoost) algorithm performed best in the internal validation process (AUC = 0.82), which was better than both the logistic regression (LR) prediction model (AUC = 0.77, *p* < 0.001) and the traditional scoring systems. Upon external validation, the XGBoost prediction model (AUC =0.81) also performed better than both the LR prediction model (AUC = 0.75, *p* = 0.03) and the traditional scoring systems. We created an online application based on the XGBoost prediction model.

**Conclusions:**

We have developed a machine learning model that has better predictive performance than traditional LR prediction models as well as other existing risk scoring systems for postoperative ARF. This model can be utilized to provide early warnings when high-risk patients are found, enabling clinicians to take prompt measures.

## Introduction

Acute aortic syndrome (AAS) is a serious and life-threatening disease process involving the ascending aorta and aortic arch. Traditionally, surgical intervention is the best way to treat AAS (1). Acute renal failure (ARF) is an important complication affecting the prognosis of AAS patients after surgery. This complication indicates that the patient has a poor prognosis, and it can increase postoperative mortality and morbidity (2). While renal replacement therapy (RRT) is a feasible treatment modality, it is arguably more important to identify the risk factors for postoperative ARF and identify potential patients with a higher likelihood of developing ARF in the postoperative setting. Some scoring systems already exist for predicting ARF after cardiac surgery (3–6), but they are usually employed for coronary artery bypass graft or heart valve surgery. Whether these scoring systems can be used in AAS-related surgery is unclear.

In recent years, machine learning has become increasingly widely used in medicine; it can help us process large amounts of data and find potential data relationships. Multiple excellent algorithms have been developed in the field of machine learning so that we can use them to build predictive models.

The main purpose of this study was to establish a predictive model for the occurrence of ARF in AAS patients after surgery through machine learning, thereby helping to identify potential patients who may develop ARF, and compare it with a traditional logistic regression (LR) prediction model and other scoring systems. This study followed the recommendations of the *Transparent Reporting of a Multivariable Prediction Model for Individual Prognosis or Diagnosis* statement (7).

## Materials and Methods

### Participants

A total of 1,637 AAS patients undergoing surgery and treatment at nine medical centers in China from January 1, 2015, to December 31, 2019 were recruited for this study. The ethics committee of Beijing Anzhen Hospital approved this retrospective cohort study (No. 2018015; Date: 2018-10-18). Patients' written informed consent was waived due to the retrospective nature of the study. We collected demographic, surgical, and clinical data with a potential relationship to the renal function of patients from admission through discharge. Patients who had renal failure before surgery, incomplete surgical data, or surgery involving the abdominal aorta and below were excluded. All patients were diagnosed with Stanford type-A AAS through aortic computed tomography angiography (CTA) by experienced imaging specialists and cardiovascular surgeons. The diagnosis of ARF was established according to the Kidney Disease: Improving Global Outcomes guidelines (8). Postoperative ARF was defined as an increase of >3 times or an increase of >4.0 mg/dL (353.6 μmol/L) in postoperative serum creatinine (Scr) or the initiation of RRT compared to baseline. The estimated glomerular filtration rate (eGFR) was calculated using the Chronic Kidney Disease Epidemiology Collaboration formula (CKD-EPI) (9). Surgery was performed by the surgical team of the medical center at the time of the patient's admission.

### Surgical Details

Anesthesia was maintained by either total intravenous anesthetics (propofol and sufentanil) or an inhalational agent (sevoflurane) with vecuronium bromide. Tranexamic acid was used for coagulation support. Cardiopulmonary bypass (CPB) was routinely instituted at 2.2 to 2.5 L/min/m^2^. When the lesion involved the aortic arch, arterial cannulation was performed in the right axillary and/or femoral artery and/or ascending aorta; venous cannulations were bicaval. Cold blood cardioplegia for myocardial protection was perfused through the left and right coronary arteries. If the distal aorta or aortic arch needed reconstruction, this process was performed under deep or moderate hypothermia and circulatory arrest. Once the distal reconstruction was complete, the aortic graft was clamped proximally. Selective anterograde perfusion was most often instituted through the innominate arteries. During core cooling, accompanying cardiac procedures, including aortic valve repair or replacement, sinus reconstruction, and root replacement, were performed if necessary. If the lesion involved only the ascending aorta, arterial cannulation was performed in the ascending aorta; venous cannulations were bicaval. Subsequently, reconstruction of the ascending aorta was performed.

### Data Pre-processing

For missing data, we used the k-nearest neighbors approach to fill in missing values (10). By calculating the Euclidean distance between each case, the missing value was imputed using the mean value from the five nearest neighbors. When the data were in the range of 0 to 1, most machine learning algorithms had excellent performance. To improve the performance of machine learning, we used the method provided by the MinMaxScaler function to scale the data during data pre-processing.

### Statistical Analysis

The description of the data and basic statistical analysis were performed using IBM SPSS Statistics for Windows Version 25.0. Continuous variables are expressed as the median (along with the first and third quartile values). Categorical variables are expressed as frequencies (n) with percentages (%). Statistical analysis of continuous variables was performed using the Mann–Whitney U test, while categorical variables were analyzed using the chi-squared test and Fisher's exact test. The area under the curve (AUC) of the receiver operating characteristic (ROC) curve was compared with the machine learning prediction model. DeLong's test (11) was used to calculate the P value. A *P* < 0.05 was considered statistically significant, and all statistical tests were two-sided.

### Establishment of Prediction Model

We used Python for programming and used the scikit-learn 0.22.1 package to build machine learning classifiers (12). The concise process of establishing and evaluating the prediction model is shown in Supplementary Figure 1.

The machine learning prediction model used data from 1,637 patients from nine medical centers in China. Among them, 1,318 patient data points from six medical centers in Beijing, Zhejiang, Shandong, Liaoning and Henan provinces were used for machine learning training and internal validation. Training and validation data were divided by ten-fold cross-validation, each time 90% of the data was used as training data, and 10% of the data was used as validation data. And 319 patient data points from three medical centers in Heilongjiang and Guangdong provinces and Xinjiang Uygur Autonomous Region were used for external validation of the prediction model. The division of internal validation and external validation data was determined by the geographic location of the medical centers. The six medical centers used for machine learning training and internal validation were located in central China, and the three medical centers used for external validation were located in northern, southern and western China. This division method was suitable for evaluating the generalization ability of predictive models.

For the selection of machine learning algorithms, we chose the support vector machine classifier (SVC) linear kernel and Nu-SVC with the radial basis function kernel in the SVC algorithm. These are two classic algorithms that use the classifier with the largest interval in the feature space for classification, and can perform data classification after linear-range or high-dimensional mapping. It is still valid when the feature has a high-dimensional relationship. Among them, SVC uses a linear algorithm, while Nu-SVC uses a radial basis function. At the same time, we chose the AdaBoost algorithm (13) and the eXtreme Gradient Boosting (XGBoost) algorithm (14) in the ensemble methods, which are popular algorithms in machine learning classifiers and can combine the predictions of several base estimators so that they have excellent performance. AdaBoost integrates multiple basic decision trees, uses misclassified data points to identify problems, and improves the model by adjusting the weights of misclassified data points. XGBoost uses negative gradients to identify problems, and calculates negative gradients to improve the model. In addition, we also tested the combination of two algorithms. This combination uses two independent algorithms to build prediction models separately, and uses the results of the two prediction models as features to retrain the new prediction model, which is also called a stacking algorithm. We tested the combination of XGBoost + random forest algorithm and XGBoost + decision tree algorithm.

### Feature Selection

To make the prediction model more accurate, we selected all demographic characteristics and preoperative clinical data as the features for machine learning. At the beginning of model training, all 134 features were used (Supplementary Table 3), and Shapley additive explanations (SHAP) was used to judge the importance of each feature. In the machine learning prediction model, SHAP can analyse the impact of each feature of each patient on the prediction result (15). Finally, the features that were considered important in all prediction models were used as the final features of the machine learning model.

### Ten-Fold Cross-Validation

Ten-fold cross-validation is considered a reliable method for model evaluation and performance improvement (16), and it was used for parameter adjustment and algorithm comparison. Since machine learning algorithms usually cannot use training data as test data, 10-fold cross-validation is generally used to evaluate machine learning algorithms. Ten-fold cross-validation can divide the data into 10 parts. The classifier used nine of them for training, and the remaining part was used for testing. Ten repetitions constituted a 10-fold cross-validation (Supplementary Figure 2). The average of 10 test results was used to evaluate the predictive ability of machine learning algorithms and parameters.

Each classifier algorithm had many parameter settings, and the choice of parameters had a great impact on the results of the classifier. We used the grid-search algorithm and internal validation data to determine the best parameters for each classifier algorithm.

The grid-search algorithm used 10-fold cross-validation to select the parameters of the machine learning algorithm. We told the grid search algorithm the potential optimal parameter range of the classifier, and the grid search algorithm used 10-fold cross validation to calculate the predictive ability of each set of parameters. After the grid-search algorithm calculated each set of parameter combinations, it told us the optimal parameter combination. After constantly changing the parameter range used by the grid search algorithm, the optimal parameter combination of this machine learning algorithm was finally obtained.

Similarly, 10-fold cross-validation was used to compare classifier algorithms. The ROC curve and AUC were calculated at each validation, and the mean and standard deviation of each AUC were compared to obtain the optimal classifier algorithm.

### Evaluation of Predictive Models

To compare the machine learning prediction model with the traditional prediction model, we used multivariable LR analysis to establish an LR prediction model with ARF as the end point. In addition, the Cleveland scoring system (3), the simplified renal index (SRI) scoring system (5) and the Leicester scoring system (6) were selected as representatives of the traditional prediction model. The endpoints of these three classic scoring systems were renal failure and RRT, and they were also scored for complex surgery. To evaluate the prediction model, we compared the machine learning prediction model with the four traditional prediction models. The ROC curve and AUC of the prediction model were calculated using internal validation data. The machine learning prediction model used 10-fold cross-validation to calculate the mean ROC curve and the AUC. In the external validation, the machine learning predictive model was trained using internal validation data. Then, we used the parametric approach based on Platt's logistic model to calibrate the probability of the machine learning model (17) and evaluated the discrimination and calibration of the model by calculating the Brier score. The trained machine learning prediction model was compared with the traditional prediction models using external validation data to evaluate the generalization ability of the prediction model.

## Results

### Patient Characteristics

A total of 1,637 patients were enrolled in the study, with 1,318 of these cases being used for machine learning training and internal validation. The main characteristics of patients in the internal validation group are presented in Table 1. The median age of the patients was 50.0 (42.0–57.0) years; 301 (22.8%) patients were female.

**Table 1:** Main characteristics of patients in the internal validation groups.

	**Overall**	**Without ARF**	**Combined ARF**	***P* value**
Number of patients (cases)	1,318	1,167	151	
Gender, female (cases)	301 (22.8%)	269 (23.1%)	32 (21.2%)	0.61
Age (years)	50.0 (42.0–57.0)	49.0 (41.0–57.0)	53.0 (46.0–60.0)	<0.001
**Information on admission**			
Pulse (beats/min)	80.0 (75.0–85.0)	80.0 (75.0–85.0)	80.0 (77.0–88.0)	0.003
Height (cm)	170.0 (166.0–175.0)	170.0 (166.0–175.0)	170.0 (165.0–175.0)	0.06
Weight (kg)	75.0 (65.0–82.4)	75.0 (65.0–83.0)	73.2 (65.0–80.0)	0.62
Body mass index (kg/m^2^)	25.4 (22.9–27.8)	25.4 (22.9–27.8)	25.4 (23.4–28.0)	0.35
Systolic pressure (mmHg)	130.0 (120.0–142.0)	130.0 (120.0–143.0)	129.0 (110.0–140.0)	0.009
Diastolic pressure (mmHg)	78.0 (70.0–84.1)	78.0 (70.0–85.0)	78.0 (70.0–82.0)	0.10
**Medical history**				
Smoking history (cases)	507 (38.5%)	449 (38.5%)	58 (38.4%)	0.99
History of previous cardiac surgery (cases)	107 (8.1%)	98 (8.4%)	9 (6.0%)	0.30
Peripheral vascular disease history (cases)	9 (0.7%)	9 (0.8%)	0 (0.0%)	0.61
**Echocardiographic results**			
Left ventricular ejection fraction (%)	63.0 (60.0–66.0)	63.0 (60.0–66.0)	62.0 (58.0–65.0)	0.21
**Preoperative laboratory examination results**			
Absolute value of leukocytes (10^9^/L)	9.69 (7.00–13.18)	9.30 (6.80–12.90)	12.33 (10.29–15.33)	<0.001
Platelets (10^9^/L)	185.0 (147.0–228.3)	189.0 (150.0–231.0)	158.0 (126.0–201.0)	<0.001
Hemoglobin (g/L)	137.0 (122.0–148.0)	137.0 (123.0–148.0)	136.0 (121.0–144.0)	0.12
CK–MB (ng/mL)	1.88 (0.90–9.30)	1.70 (0.80–8.20)	7.10 (1.60–15.00)	<0.001
Lactate dehydrogenase (U/L)	221.5 (179.0–288.3)	214.0 (176.0–279.0)	277.0 (225.0–332.2)	<0.001
D–dimer (ng/mL)	1,100.0 (270.8–3,269.3)	940.0 (231.0–2,887.0)	3,328.0 (1,120.0–14,485.0)	<0.001
INR	31.9 (28.9–36.5)	31.9 (28.8–36.3)	32.2 (29.5–37.8)	0.02
APTT (s)	48.8 (39.6–60.1)	48.6 (39.5–60.0)	51.4 (40.5–65.1)	0.03
Blood amylase (U/dL)	21.0 (15.0–34.0)	21.0 (15.0–33.0)	27.0 (16.0–45.0)	0.07
ALT (U/mL)	22.0 (18.0–32.0)	22.0 (17.0–30.0)	29.0 (21.0–46.0)	0.005
AST (U/mL)	39.1 (35.6–42.1)	39.2 (35.6–42.2)	38.9 (35.2–40.7)	<0.001
Albumin (g/mL)	78.3 (64.6–99.6)	76.7 (63.9–95.8)	99.7 (78.0–138.6)	0.08
Creatinine (μmol/L)	6.30 (4.99–8.10)	6.10 (4.90–7.78)	8.20 (6.01–10.30)	<0.001
BUN (mmol/mL)	95.0 (73.1–107.1)	97.1 (77.0–108.4)	69.1 (49.0–93.4)	<0.001
eGFR (ml/min/1.73 m^2^)	6.49 (5.39–7.77)	6.38 (5.30–7.67)	7.26 (6.28–8.48)	<0.001
Fasting blood glucose (mmol/L)	9.69 (7.00–13.18)	9.30 (6.80–12.90)	12.33 (10.29–15.33)	<0.001
**Diagnosis**				
Coronary artery disease (cases)	35 (2.7%)	33 (2.8%)	2 (1.3%)	0.42
Congestive heart failure (cases)	26 (2.0%)	23 (2.0%)	3 (2.0%)	1.00
Chronic respiratory disease (cases)	31 (2.4%)	28 (2.4%)	3 (2.0%)	1.00
Hypertension (cases)	905 (68.7%)	786 (67.4%)	119 (78.8%)	0.004
Diabetes (cases)	62 (4.7%)	54 (4.6%)	8 (5.3%)	0.71
**Surgery**				
Operative duration (min)	405.0 (340.0–479.0)	396.0 (330.0–465.0)	454.6 (390.0–520.0)	<0.001
Emergency surgery (cases)	650 (49.3%)	536 (45.9%)	114 (75.5%)	<0.001
Cardiopulmonary bypass time (min)	186.0 (144.0–224.0)	181.0 (140.0–218.0)	224.0 (188.0–266.0)	<0.001
Aortic cross–clamp time (min)	104.0 (82.6–131.0)	102.0 (80.0–127.0)	125.0 (103.0–149.0)	<0.001
With circulatory arrest (cases)	997 (75.6%)	855 (73.3%)	142 (94.0%)	<0.001
Circulatory arrest time (min)	21.0 (17.4–27.0)	21.0 (17.6–26.6)	21.0 (17.0–27.0)	0.88
Nasopharyngeal temperature when circulatory arrest (°C)	24.1 (23.1–24.9)	24.1 (23.2–24.9)	23.5 (22.5–24.5)	<0.001
Rectal temperature when circulatory arrest (°C)	25.6 (24.8–26.7)	25.8 (24.9–26.8)	25.0 (24.0–26.0)	<0.001
RBC transfusion volume (U)	4.00 (0.00–6.00)	3.50 (0.00–6.00)	5.50 (4.00–8.00)	<0.001

*INR, international normalized ratio; APTT, activated partial thromboplastin time; ALT, alanine aminotransferase; AST, aspartate transaminase; BUN, blood urea nitrogen; eGFR, estimated glomerular filtration rate*.

### Incidence and Prognosis of Postoperative ARF

The incidence of ARF after aortic surgery was 11.5% (151 in 1,318). The prognostic characteristics of the patients are presented in Supplementary Table 1. Patients with postoperative ARF had a poor prognosis and had longer ICU stays (204.0 (104.5–308.2) h vs. 43.0 (20.0–112.5) h, *P* < 0.001) as well as longer ventilator use times (114.0 (62.0–179.0) h vs. 20.0 (15.0–48.0) h, *P* < 0.001). Postoperative ARF may be related to the use of more blood products and drug infusions. Furthermore, patients with postoperative ARF had more postoperative complications (74.8 vs. 34.5%, *P* < 0.001). Most importantly, there were significant differences in mortality between patients with and without ARF (12.6 vs. 0.8%, respectively, *P* < 0.001).

### Risk Factors for Postoperative ARF

As a comparison with machine learning models, we used traditional statistical methods to analyse all preoperative and intraoperative factors that either had significant differences or were clinically believed to be related to ARF and calculated the risk factors for postoperative ARF. A multivariable binary LR with the “Forward: LR” method was conducted to determine the risk factors for postoperative ARF (Table 2). The results showed that, among the preoperative factors, older age, a higher pulse rate, emergency surgery, and an increased absolute value of leukocytes in the preoperative setting were all risk factors. It was also noted that an increased estimated glomerular filtration rate (eGFR) and platelet count were protective factors against postoperative ARF. In the combined analysis of preoperative and intraoperative factors, in addition to the aforementioned preoperative factors, longer cardiopulmonary bypass time, lower rectal temperature when circulatory arrest, and surgery with circulatory arrest were risk factors for postoperative ARF. We used preoperative factors to establish an LR prediction model for the predictive model to have the ability to predict postoperative ARF of patients before surgery (Table 2).

**Table 2:** Multivariable binary logistic regression results.

**Characteristics**	**B**	**Standard error**	***P* value**	**OR value**	**OR 95%CI**
**Preoperative factors only**					
Age (years)	0.028	0.010	0.003	1.029	1.010–1.048
Absolute value of leukocyte	0.076	0.022	0.001	1.079	1.033–1.127
Pulse (beats/min)	0.021	0.008	0.006	1.021	1.006–1.037
eGFR (ml/min/1.73m^2^)	−0.019	0.004	<0.001	0.982	0.974–0.989
Platelet (10^9^/L)	−0.005	0.002	0.003	0.995	0.992–0.998
Emergency surgery	0.779	0.216	<0.001	2.179	1.426–3.331
Constant	−4.207	1.057	<0.001	0.015	
**Preoperative and intraoperative factors**					
Age (years)	0.032	0.010	0.001	1.032	1.012–1.052
Absolute value of leukocyte	0.062	0.023	0.008	1.064	1.017–1.114
Pulse (beats/min)	0.023	0.008	0.004	1.023	1.007–1.039
eGFR (ml/min/1.73m^2^)	−0.017	0.004	<0.001	0.983	0.976–0.991
Platelet (10^9^/L)	−0.004	0.002	0.011	0.996	0.993–0.999
Emergency surgery	0.587	0.222	0.008	1.799	1.164–2.781
Cardiopulmonary bypass time (min)	0.006	0.002	<0.001	1.006	1.003–1.010
Surgery with circulatory arrest	0.875	0.379	0.021	2.400	1.142–5.042
Rectal temperature when circulatory arrest (°C)	−0.129	0.054	0.017	0.879	0.792–0.977
Constant	−3.315	1.842	0.072	0.036	

*eGFR, Estimated Glomerular Filtration Rate*.

### Machine Learning Prediction Model

#### Feature Selection

In the initial stage, we built machine learning models using all the preoperative features and unoptimized parameters. We analyzed the feature importance of these machine learning models through SHAP (15) and finally selected 15 features for building machine learning prediction models (Table 3).

**Table 3:** Features used to build machine learning prediction model.

**Information on admission**	
	Age (years)
	Pulse (beats/min)
	BMI (kg/m^2^)
	Diastolic pressure (mmHg)
**Echocardiographic results**	
	Left ventricular ejection fraction (%)
**Preoperative laboratory examination results**	
	Absolute value of leukocytes (10^9^/L)
	Platelets (10^9^/L)
	D-dimer (ng/mL)
	INR
	APTT (s)
	ALT (U/mL)
	Albumin (g/mL)
	eGFR (ml/min/1.73 m^2^)
	Fasting blood glucose (mmol/L)
**Surgery**	
	Emergency surgery

*BMI, body mass index; INR, international normalized ratio; APTT, activated partial thromboplastin time; ALT, alanine aminotransferase; eGFR, estimated glomerular filtration rate*.

Among the 15 features used to establish these models, we collected complete demographic and renal function data. However, as AAS patients may require emergency surgery, occasionally, the blood test results were partially missing. We used the k-nearest neighbors approach to fill in missing values. Supplementary Table 2 shows the details of missing values.

#### Internal Validation

**Figure 1:**
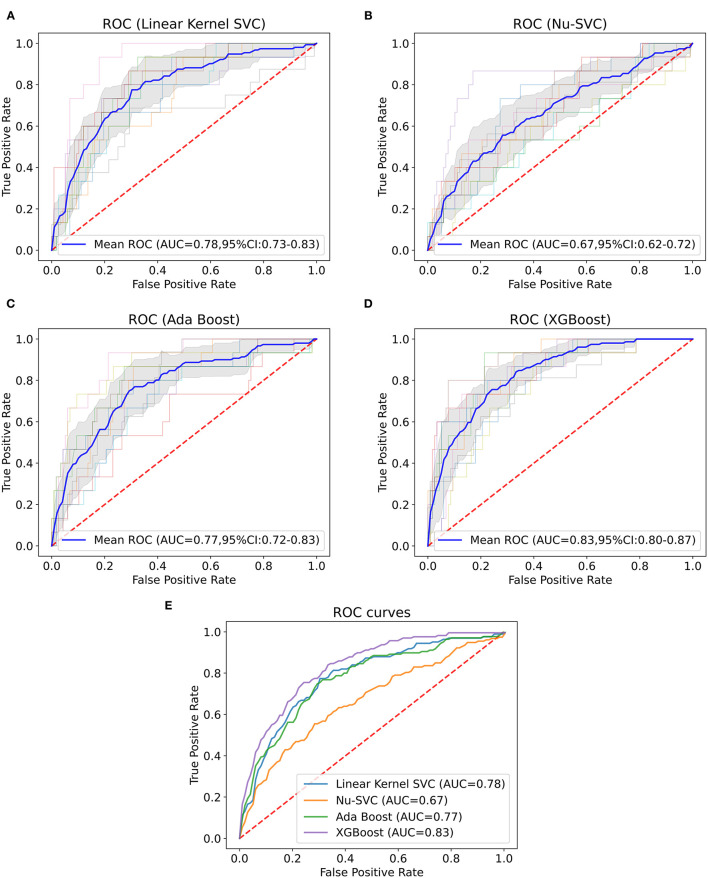
Mean ROC curve and AUC of machine learning models. This figure depicts the mean ROC curve and AUC of the linear kernel SVC **(A)**, Nu-SVC **(B)**, AdaBoost **(C)** and XGBoost **(D)** using internal validation data (*n* = 1,318). The blue line represents the mean of each ROC curve after 10-fold cross-validation. The shaded area is the 95% confidence interval of the mean ROC curve. The other translucent lines are ROC curves for each cross-validation. **(E)** Comparison of the mean ROC curves for each algorithm.

Machine learning models were trained using internal validation data, and the performance of the machine learning models was evaluated using 10-fold cross-validation. The results showed the mean ROC curve and AUC of each machine learning model after 10-fold cross-validation (Figure 1). We found that among the prediction models established by a single algorithm, the XGBoost machine learning model performed best (AUC = 0.82, 95% confidence interval (CI): 0.79–0.85), and the combination of XGBoost and other algorithms did not improve performance (Supplementary Figure 3); thus, we chose the XGBoost model as the final machine learning model to evaluate its performance. This model had 750 gradient boosted trees, the maximum tree depth was eight, the learning rate was 0.01, the subsample ratio of columns when constructing each tree was 0.75, and the subsample ratio of the training instance was 0.68.

**Figure 2:**
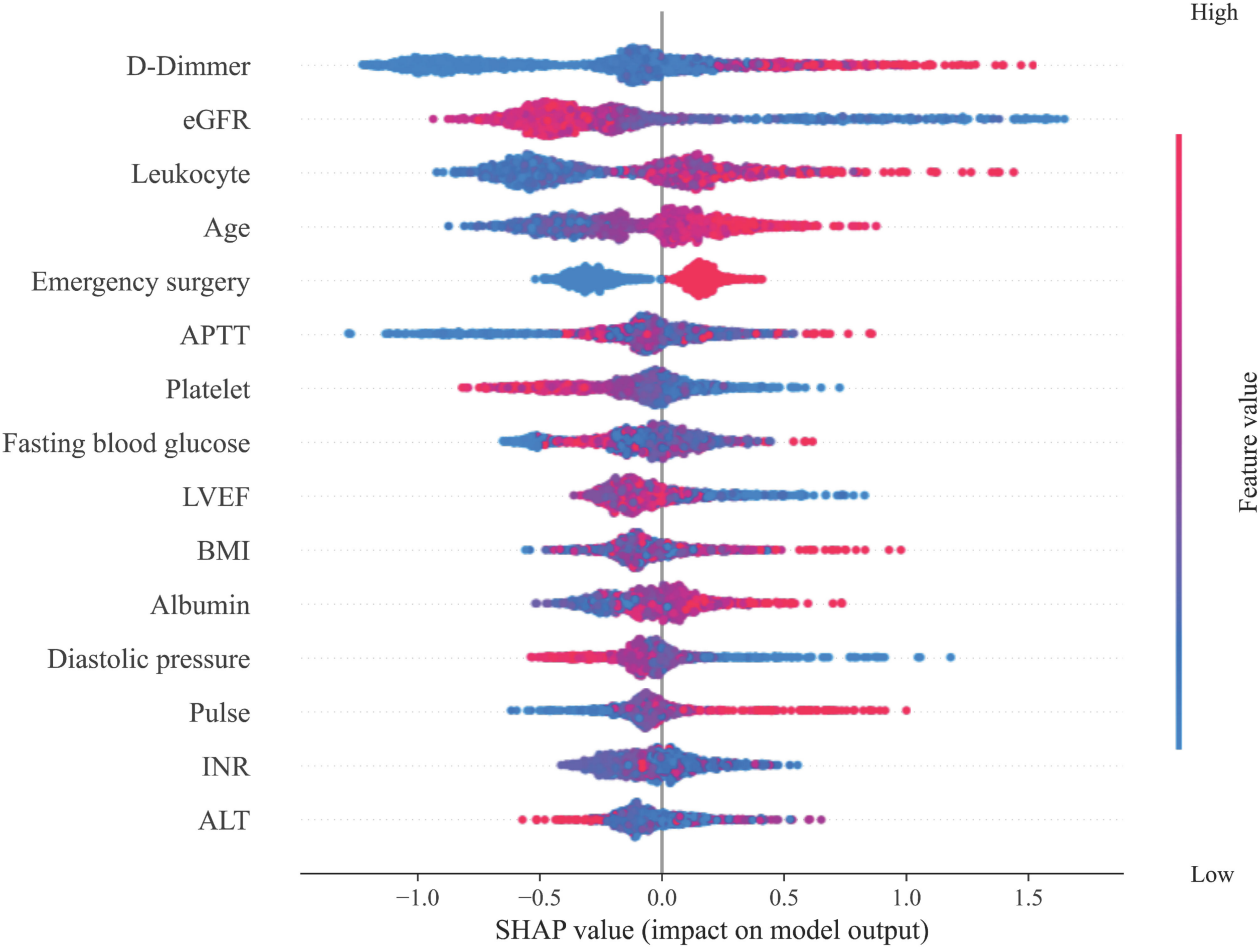
Feature importance analysis. This figure shows the results of the analysis on the importance of the features in the XGBoost model through the SHAP method. Each feature value of each patient is marked as a dot on the graph. The color of the dot represents the degree of deviation of the feature value from the overall value according to the ordinate, and purple represents that the feature of the patient is close to the mean of the feature of the overall patient value. The SHAP value of the dot indicates the influence of the feature on the prediction result. A negative SHAP value indicates that the patient's risk of ARF is reduced, while a positive SHAP value indicates that the patient's risk of ARF is increased.

Subsequently, the importance of each feature of the XGBoost model was analyzed by the SHAP method. Figure 2 shows the results of the feature importance analysis, with more important features distributed on the top and relatively unimportant features on the bottom. Most of the characteristics, either positively or negatively, correlated with the prediction results; however, activated partial thromboplastin time (APTT), fasting blood glucose, body mass index (BMI), international normalized ratio (INR), and alanine aminotransferase (ALT) that were either too high or too low increased the risk of ARF. At the same time, we also analyzed the feature importance based on the fitted trees of the XGBoost model (Supplementary Figure 4), and the result is similar to the result of SHAP.

**Figure 3:**
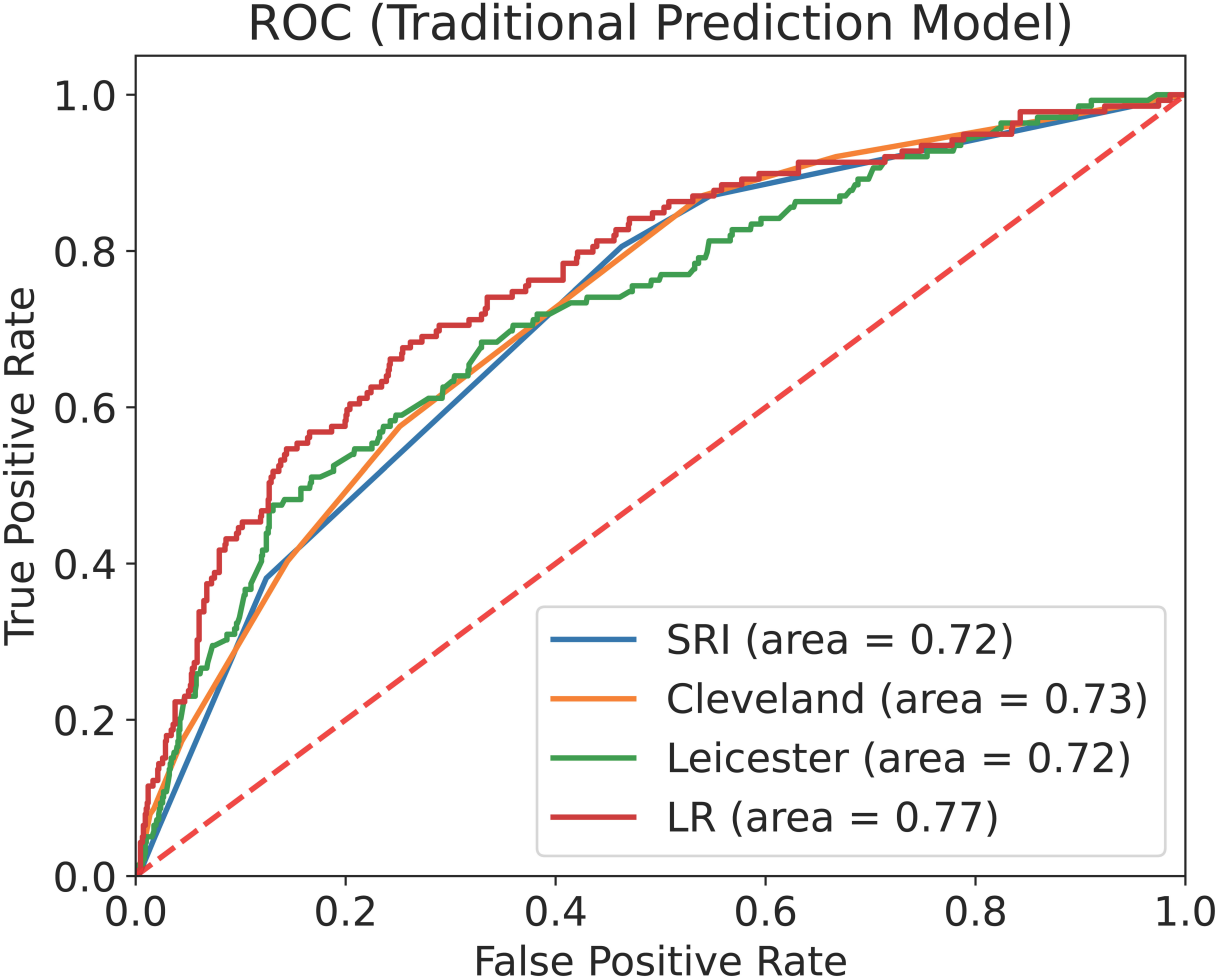
ROC curve and AUC of the traditional prediction models with internal validation data. This figure describes the ROC curve and the AUC of the Cleveland scoring system, the SRI scoring system, the Leicester scoring system and the LR prediction model with internal validation data (*n* = 1,318).

We used internal validation data to calculate the ROC curve and the AUC of the four traditional prediction models (Figure 3) and found that the XGBoost model (AUC = 0.82, 95% CI: 0.79–0.85) performed better than the LR prediction model (AUC = 0.77, 95% CI: 0.73–0.81, P <0.001), the Cleveland scoring system (AUC = 0.73, 95% CI: 0.69–0.77, P <0.001), the SRI scoring system (AUC = 0.72, 95% CI: 0.68–0.76, P <0.001), and the Leicester scoring system (AUC = 0.72, 95% CI: 0.68–0.77, *P* < 0.001) (Table 4).

**Table 4:** Performance of machine learning prediction model and scoring system.

	**Prediction methods**	**AUC**	**95% CI of AUC**	***P value***
Internal validation				
	Machine learning prediction model (XGBoost)	0.82	0.79–0.85	
	LR prediction model	0.77	0.73–0.81	<0.001
	Cleveland scoring system	0.73	0.69–0.77	<0.001
	SRI scoring system	0.72	0.68–0.76	<0.001
	Leicester scoring system	0.72	0.68–0.77	<0.001
External validation				
	Machine learning prediction model (XGBoost)	0.81	0.75–0.88	
	LR prediction model	0.75	0.67–0.83	0.03
	Cleveland scoring system	0.71	0.63–0.80	0.04
	SRI scoring system	0.70	0.61–0.79	0.02
	Leicester scoring system	0.67	0.59–0.75	0.002

*AUC, area under the receiver operating characteristic (ROC) curve; XGBoost, eXtreme Gradient Boosting; LR, logistic regression; SRI, simplified renal index*.

*The AUC of the ROC curve was compared with a machine learning prediction model (XGBoost), and DeLong's test was used to calculate the P value*.

#### External Validation

The external validation group included 319 patients. The comparison of the internal and external validation group characteristics is presented in Table 5. The median of the average age of patients in the external validation group was 50.0 (16.0) years. The incidence of ARF after aortic surgery was similar to that in the internal validation group (11.0 vs. 11.5%, *P* = 0.807).

**Table 5:** Comparison of characteristics in the internal and external validation groups.

	**Internal validation group**	**External validation group**	***P* value**
Number of patients (cases)	1,318	319	
Incidence of postoperative ARF	151 (11.5%)	35 (11.0%)	0.81
Need CRRT treatment (cases)	137 (90.7%)	30 (85.7%)	0.36
**Information on admission**			
Gender, female (cases)	301 (22.8%)	65 (20.4%)	0.34
Age (years)	50.0 (42.0–57.0)	50.0 (43.0–59.0)	0.10
Pulse (beats/min)	80.0 (75.0–85.0)	80.0 (74.0–85.0)	0.62
Height (cm)	170.0 (166.0–175.0)	170.0 (165.0–175.0)	0.59
Weight (kg)	75.0 (65.0–82.4)	74.0 (65.0–83.0)	0.42
Body mass index (kg/m^2^)	25.4 (22.9–27.8)	25.4 (23.1–27.8)	0.71
Systolic pressure (mmHg)	130.0 (120.0–142.0)	135.0 (120.0–160.0)	<0.001
Diastolic pressure (mmHg)	78.0 (70.0–84.1)	80.0 (70.0–95.0)	<0.001
**Medical history**			
Smoking history (cases)	507 (38.5%)	96 (30.1%)	0.005
History of previous cardiac surgery (cases)	107 (8.1%)	20 (6.3%)	0.27
Peripheral vascular disease history (cases)	9 (0.7%)	3 (0.9%)	0.71
**Echocardiographic results**			
Left ventricular ejection fraction (%)	63.0 (60.0–66.0)	61.0 (57.0–66.0)	0.02
**Preoperative laboratory examination results**		
Absolute value of leukocytes (10^9^/L)	9.69 (7.00–13.18)	11.08 (7.78–14.29)	<0.001
Platelets (10^9^/L)	185.0 (147.0–228.3)	184.0 (143.0–236.0)	0.93
Hemoglobin (g/L)	137.0 (122.0–148.0)	134.0 (122.0–147.0)	0.19
CK-MB (ng/mL)	1.88 (0.90–9.30)	3.30 (1.00–11.60)	<0.001
Lactate dehydrogenase (U/L)	221.5 (179.0–288.3)	224.0 (189.0–277.4)	0.32
D-dimer (ng/mL)	1,100.0 (270.8–3,269.3)	715.9 (18.4–4,230.0)	<0.001
INR	31.9 (28.9–36.5)	34.0 (29.1–39.7)	0.39
APTT (s)	48.8 (39.6–60.1)	50.4 (43.0–58.4)	<0.001
Blood amylase (U/dL)	21.0 (15.0–34.0)	23.0 (15.0–36.0)	0.14
ALT (U/mL)	22.0 (18.0–32.0)	22.0 (18.0–33.0)	0.14
AST (U/mL)	39.1 (35.6–42.1)	38.5 (35.0–42.0)	0.56
Albumin (g/mL)	78.3 (64.6–99.6)	80.4 (66.8–101.6)	0.12
Creatinine (μmol/L)	6.30 (4.99–8.10)	6.40 (5.10–8.70)	0.48
BUN (mmol/mL)	95.0 (73.1–107.1)	92.3 (70.4–107.3)	0.05
eGFR (ml/min/1.73 m^2^)	6.49 (5.39–7.77)	6.63 (5.40–7.65)	0.22
Fasting blood glucose (mmol/L)	9.69 (7.00–13.18)	11.08 (7.78–14.29)	0.94
**Diagnosis**			
Coronary artery disease (cases)	35 (2.7%)	6 (1.9%)	0.43
Congestive heart failure (cases)	26 (2.0%)	1 (0.3%)	0.04
Chronic respiratory disease (cases)	31 (2.4%)	15 (4.7%)	0.02
Hypertension (cases)	905 (68.7%)	216 (67.7%)	0.74
Diabetes (cases)	62 (4.7%)	13 (4.1%)	0.63
**Surgery**			
Operative duration (min)	405.0 (340.0–479.0)	390.0 (321.2–480.0)	0.21
Emergency surgery (cases)	650 (49.3%)	164 (51.4%)	0.50
Cardiopulmonary bypass time (min)	186.0 (144.0–224.0)	184.0 (135.2–236.0)	0.70
Aortic cross-clamp time (min)	104.0 (82.6–131.0)	108.0 (82.0–140.0)	0.13
With circulatory arrest (cases)	997 (75.6%)	223 (69.9%)	0.04
Circulatory arrest time (min)	21.0 (17.4–27.0)	21.2 (17.0–28.0)	0.63
Nasopharyngeal temperature when circulatory arrest (°C)	24.1 (23.1–24.9)	24.2 (23.0–25.0)	0.32
Rectal temperature when circulatory arrest (°C)	25.6 (24.8–26.7)	25.8 (24.5–27.0)	0.84
RBC transfusion volume (U)	4.00 (0.00–6.00)	4.00 (2.00–6.00)	0.006

*CRRT, continuous renal replacement therapy; INR, international normalized ratio; APTT, activated partial thromboplastin time; ALT, alanine aminotransferase; AST, aspartate transaminase; BUN, blood urea nitrogen; eGFR, estimated glomerular filtration rate*.

After probability calibration, the Brier score of the machine learning prediction model using the external validation data was 0.087, which showed that the prediction model had good discrimination and calibration. Using external validation data for evaluation, we found that the XGBoost model after probability calibration (AUC = 0.81, 95% CI: 0.75–0.88) performed better than the LR prediction model (AUC = 0.75, 95% CI: 0.67–0.83, *P* = 0.03), the Cleveland scoring system (AUC = 0.71, 95% CI: 0.63–0.80, *P* = 0.04), the SRI scoring system (AUC = 0.70, 95% CI: 0.61–0.79, *P* = 0.02), and the Leicester scoring system (AUC = 0.67, 95% CI: 0.59–0.75, *P* = 0.002) (Table 4).

Finally, to make the XGBoost prediction model easy to use, we developed an application (https://ljzyal.github.io/ARF/) for clinical use. The application used a probability-calibrated XGBoost prediction model, which had the same performance as the prediction model in external validation. We set the cut-off value based on the results of external validation. The prediction model had a sensitivity of 82.9% and a specificity of 67.6%. The risk calculated by the application increased with the possibility of postoperative ARF.

## Discussion

### Factors Influencing Postoperative ARF and the Role of a Prediction Model

ARF is the end stage of acute kidney injury, and it is the most common major complication following cardiac surgery (18). ARF can lead to poor patient prognosis and is independently associated with increased morbidity and mortality after cardiac surgery (19). In this study, the incidence of postoperative ARF in AAS patients reached 11.5%, and patients with ARF had a longer ICU length of stay, longer ventilator use time and a worse prognosis.

The mechanism of ARF after cardiac surgery remains to be elucidated, and its pathogenesis is currently thought to be related to renal hypoperfusion, tissue ischaemia-reperfusion injury and the inflammatory response (20). Previous studies have shown that risk factors for ARF include female sex, advanced age, previous heart surgery, chronic obstructive pulmonary disease, diabetes, complex heart surgery, prolonged cardiopulmonary bypass, rapid heart rate, emergency surgery, and intraoperative infusion of 2 or more packed red blood cell (RBC) units (21–23). In this study, we found that patients with AAS have more complex risk factors for postoperative ARF. In addition to the above factors, we found that postoperative ARF was also related to preoperative leukocyte and platelet counts, which may be because the occurrence of ARF is related to the inflammatory response (20). The higher preoperative leukocyte count may indicate that the inflammatory response caused by AAS is more serious. This effect may continue to play a role after surgery, making postoperative ARF more likely to occur. Furthermore, abnormal blood coagulation is another potential mechanism of ARF (24). Lower preoperative platelets may be a manifestation of a hypercoagulable state and intravascular coagulation, which makes AAS patients with higher preoperative platelet counts less prone to postoperative ARF.

Early identification of patients with a higher ARF risk can help clinicians strengthen patient monitoring and take measures to prevent ARF. Many studies have used risk factors or novel biomarkers to build prediction models for ARF (3–6, 25). Novel biomarker-related prediction methods, however, are usually cumbersome, and no new biomarker has been widely accepted (25). Currently, the best-performing large-sample model is poor at predicting ARF after complex surgery (6). Aortic surgery usually results in a higher incidence of postoperative ARF; therefore, postoperative ARF prediction methods for complex heart and aortic surgeries are necessary.

### Clinical Applications and Strategies

According to the results of this study, we recommend the following measures to reduce the occurrence of ARF. Once the model is used to predict the postoperative risk of ARF, for those with a low predicted risk, it is recommended to perform surgery in a timely fashion once the patient is surgically prepared.

For patients with a higher predicted risk of ARF, it is recommended to attempt to ameliorate the modifiable risk factors that are included in the prediction model. This could be achieved by improving preoperative preparations, such as administering antimicrobials, considering platelet transfusion, controlling blood glucose levels, maintaining adequate diastolic blood pressure, and controlling heart rate. In addition, during the operation, it is recommended to pay more attention to renal function and to maintain renal perfusion by taking measures to maintain circulatory stability. In addition to the aforementioned modifications, intraoperative innovations in surgical methods should be adopted, which can help reduce the operative and CPB time.

Additionally, for patients noted to be at a higher risk of ARF, it is also recommended to use stricter monitoring and more favorable preventive and treatment measures in perioperative management, which could include minimizing the use of nephrotoxic drugs and administering treatment for renal injury as soon as possible to prevent patients from progressing to ARF.

### Features of Machine Learning Prediction Models

Risk prediction plays an important role in cardiovascular disease research. As the most commonly used traditional predictive model, LR sometimes cannot handle complex clinical data and thus cannot obtain an ideal predictive model. Conversely, machine learning can handle complex clinical data and thus potentially has more advantages (26). In this study, by selecting AAS as a disease process for focused research, we found that the performance of machine learning predictive models is better than that of traditional predictive models. This suggests that machine learning algorithms are more suitable for building clinical prediction models and have a higher performance than LR.

In this study, we found that the XGBoost algorithm has the best prediction performance and still has excellent performance in external validation. XGBoost is a machine learning algorithm that uses classification and regression trees as weak classifiers (14). Compared with other algorithms, the XGBoost algorithm allows easy adjustment of parameters and can deal with nonlinear features. It usually has higher sensitivity and specificity when overfitting is avoided. In most cases, XGBoost has higher prediction performance than other algorithms (27, 28).

Machine learning algorithms are also suitable for the construction of other predictive models, which have excellent performance and can be continuously trained with new data to have greater potential. After the prediction model is established, the newly collected data can be used to continue training, thereby enhancing the generalization ability of the prediction model.

Machine learning algorithms have been considered a black box in the past, which is the main disadvantage compared to LR. However, the SHAP method can explain the machine learning prediction model. We used the SHAP method to analyse the importance of particular features in the prediction model. This method can analyse the impact of each variable on each patient so the prediction model is interpretable. We found that the SHAP method is effective in determining the importance of particular individual features.

To compare with LR, all features included in an LR are also considered important features in machine learning prediction models. Concurrently, however, the SHAP method also judges other features, those that are not considered statistically significant in the LR, as important features. This possibly results in LR not including certain relevant features, whereas the SHAP method does not exclude such features. According to the results of the feature importance analysis, we can also judge the impact of each variable on the results to determine the patient's treatment direction to prevent postoperative ARF.

### Limitations

First, all data for this study were sourced from China. Due to ethnic differences, the performance of our predictive model in other countries may decrease. However, our research method is innovative, and it is feasible to establish such a model in other countries through this investigational method. Second, machine learning algorithms are more complex than LR, and model representation is also very complicated (29). Our predictive model cannot be similar to LR, and it does not provide a scoring system for clinicians. We have therefore developed an online application for convenience. Third, our machine learning prediction model needs more extensive data for verification. Finally, although the performance of our prediction model was better than that of LR, some data were not involved in the initial data collection, such as detailed laboratory test results, detailed medical history and detailed documentation of the use of nephrotoxic drugs. Supervised machine learning can improve the model after supplementing these data, and consequently, our predictive model has the potential to improve.

In summary, our findings suggest that machine learning prediction models can provide better prediction performance than traditional LR prediction models and other existing risk scoring systems for AAS and complex cardiac and aortic surgeries. This predictive model is helpful for the early detection of patients with high ARF risk, thus enabling clinicians to take early measures to prevent and treat ARF.

## Data Availability Statement

The raw data supporting the conclusions of this article will be made available by the authors, without undue reservation.

## Ethics Statement

The studies involving human participants were reviewed and approved by the Ethics Committee of Beijing Anzhen Hospital. Written informed consent for participation was not required for this study in accordance with the national legislation and the institutional requirements.

## Author Contributions

HZ and WJ designed the research. JL and MG analyzed the data and wrote the paper. YJ analyzed the data and revised the manuscript. LS, LH, RF, TG, ZZ, CZ, GZ, XQ, CQ, and YC were responsible for the data collection. All authors read and approved the final manuscript.

## Funding

This study was supported by the National Key Research and Development Program of China (2017YFC1308000), Capital Health Development Research Project (2018-2-2066 and 2018-4-2068), National Science Foundation of China (81600362 and 81800404), Beijing Lab for Cardiovascular Precision Medicine (PXM2017_014226_000037), Beijing Advanced Innovation Center for Big Data-based Precision Medicine (PXM2021_014226_000026), Beijing Municipal Administration of Hospitals' Youth Program (QML20180601), and Foundation of Beijing Outstanding Young Talent Training Program (2017000021469G254). The funders did not have a role in the study design, collection, analysis or reporting of data, preparation of the manuscript or decision to submit for publication.

## Conflict of Interest

The authors declare that the research was conducted in the absence of any commercial or financial relationships that could be construed as a potential conflict of interest.

## Publisher's Note

All claims expressed in this article are solely those of the authors and do not necessarily represent those of their affiliated organizations, or those of the publisher, the editors and the reviewers. Any product that may be evaluated in this article, or claim that may be made by its manufacturer, is not guaranteed or endorsed by the publisher.
