# Supplementary material for: Machine Learning Prediction Model for Acute Renal Failure After Acute Aortic Syndrome Surgery
Source: Front Med (Lausanne). 2022 Jan 17;8:728521. doi: 10.3389/fmed.2021.728521 (PMC8801502; doi:10.3389/fmed.2021.728521)
Supplement: Supplementary file 1 [file Data_Sheet_1.docx]

Supplementary Material

# Supplementary Figures and Tables

## Supplementary Figures

##
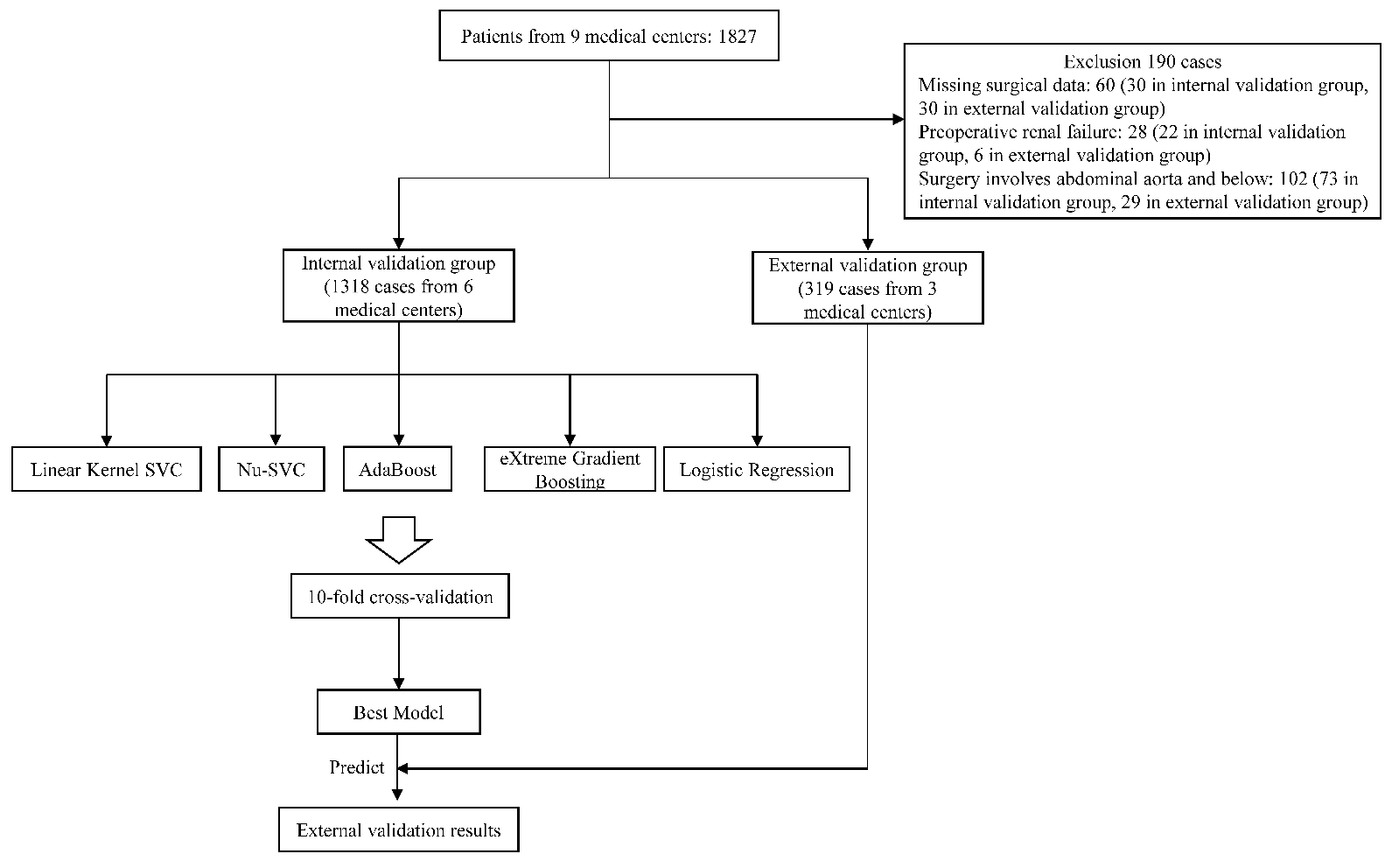


**Supplementary Figure 1 Concise flow chart for establishing and evaluating the prediction model.** Supplementary Figure 1 depicts the concise process of establishing and evaluating a prediction model.





**Supplementary Figure 2 Schematic diagram of 10-fold cross-validation.** The internal validation data are randomly divided into ten equal parts, one of which is selected as test data each time, and the other data are used as training data. After the model is trained using the training data, the test data are used to obtain the ROC curve. After ten repetitions, the average ROC curve was calculated as the basis for evaluating the predictive ability of the model.


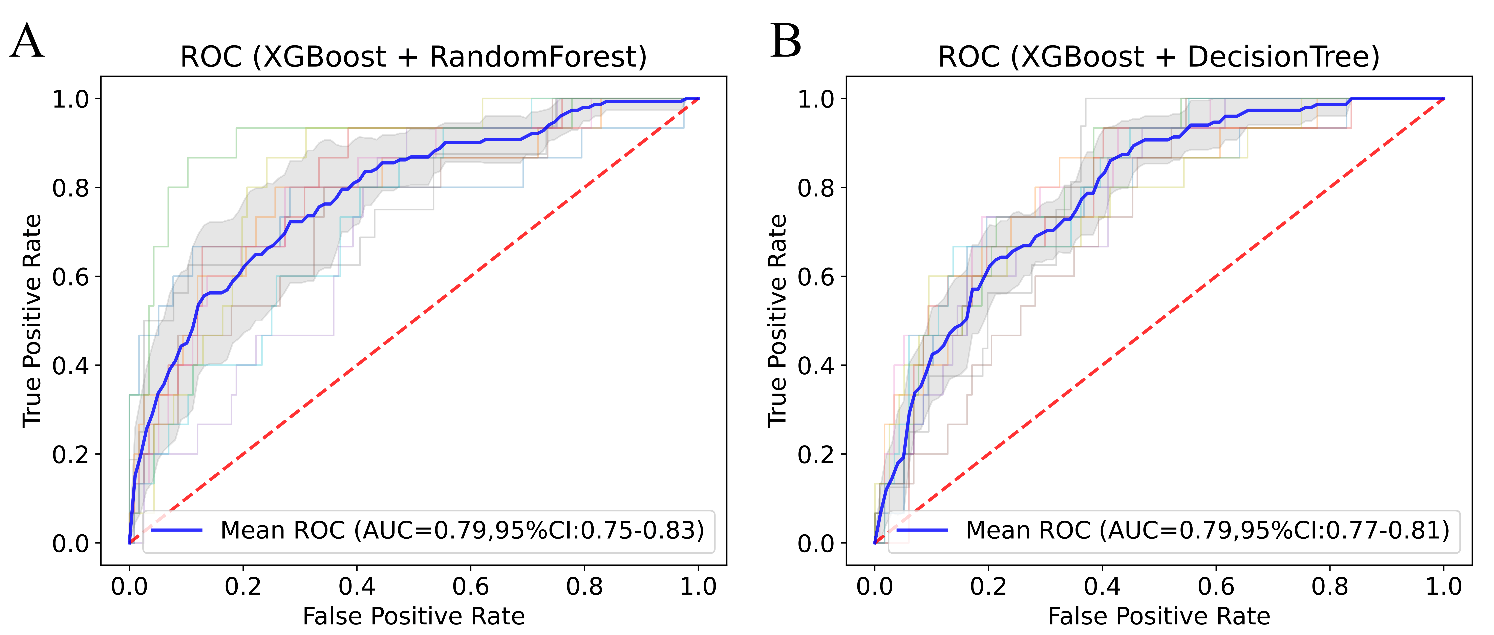


**Supplementary Figure 3 Mean ROC curve and AUC of combined models.** Supplementary Figure 3 depicts the mean ROC curve and AUC of XGBoost + random forest (A) and XGBoost + decision tree (B) using internal validation data (n = 1318). The blue line represents the mean of each ROC curve after 10-fold cross-validation. The shaded area is the 95% confidence interval of the mean ROC curve. The other translucent lines are ROC curves for each cross-validation.


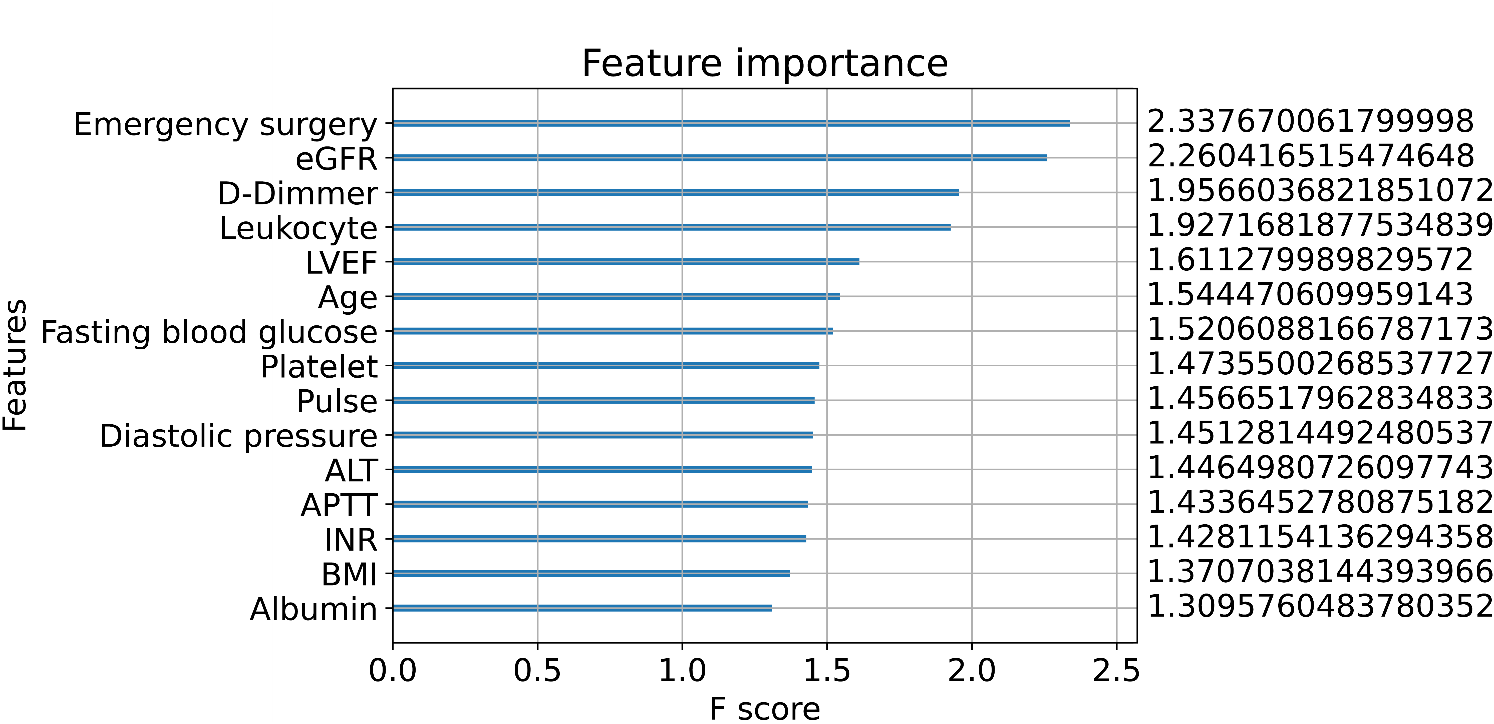


**Supplementary Figure 4 Feature importance of XGBoost model.** Supplementary Figure 4 depicts the importance of each feature of the XGBoost model. Features with higher scores were more important.

## Supplementary Tables

| **Supplementary Table 1 Prognosis Characteristics of the Patients in the Internal Validation Groups** | | | | |
| --- | --- | --- | --- | --- |
|  | Overall (n=1318) | Without ARF (n=1167) | Combined ARF (n=151) | P value |
| **ICU treatment** |  |  |  |  |
| ICU stays (hours) | 57.0 (21.0-137.0) | 43.0 (20.0-112.5) | 204.0 (104.5-308.2) | < 0.001 |
| Postoperative awareness recovery time (hours) | 5.00 (3.00-10.00) | 5.00 (3.00-9.00) | 8.00 (4.00-36.00) | < 0.001 |
| Ventilator use time (hours) | 23.7 (16.0-69.5) | 20.0 (15.0-48.0) | 114.0 (62.0-179.0) | < 0.001 |
| Endotracheal intubation again (case) | 79 (6.0%) | 51 (4.4%) | 28 (18.5%) | < 0.001 |
| Tracheotomy (case) | 26 (2.0%) | 15 (1.3%) | 11 (7.3%) | < 0.001 |
| Total RBC transfusion volume (U) | 4.00 (0.00-7.85) | 4.00 (0.00-6.00) | 8.00 (4.00-14.00) | < 0.001 |
| Total plasma transfusion volume (mL) | 240.0 (0.0-550.0) | 160.0 (0.0-450.0) | 520.0 (400.0-800.0) | < 0.001 |
| Total platelet transfusion volume (U) | 0.00 (0.00-2.00) | 0.00 (0.00-1.20) | 2.00 (0.20-5.00) | < 0.001 |
| Total cryoprecipitation transfusion volume (U) | 0.00 (0.00-2.00) | 0.00 (0.00-0.00) | 0.00 (0.00-10.00) | < 0.001 |
| With pituitrin used (case) | 51 (3.9%) | 32 (2.7%) | 19 (12.6%) | < 0.001 |
| With protamine used (case) | 523 (39.7%) | 436 (37.4%) | 87 (57.6%) | < 0.001 |
| With fibrinogen used (case) | 279 (21.2%) | 237 (20.3%) | 42 (27.8%) | 0.03 |
| With factor Ⅶ used (case) | 45 (3.4%) | 32 (2.7%) | 13 (8.6%) | < 0.001 |
| **Postoperative complications (case)** | 516 (39.2%) | 403 (34.5%) | 113 (74.8%) | < 0.001 |
| Neurological complications (case) | 93 (7.1%) | 54 (4.6%) | 39 (25.8%) | < 0.001 |
| Visceral complications (case) | 46 (3.5%) | 8 (0.7%) | 38 (25.2%) | < 0.001 |
| Liver failure (case) | 22 (1.7%) | 3 (0.3%) | 19 (12.6%) | < 0.001 |
| Stress ulcers (case) | 32 (2.4%) | 19 (1.6%) | 13 (8.6%) | < 0.001 |
| Ischemic complications (case) | 17 (1.3%) | 8 (0.7%) | 9 (6.0%) | < 0.001 |
| Cardiac complications (case) | 62 (4.7%) | 33 (2.8%) | 29 (19.2%) | < 0.001 |
| Low cardiac output syndrome (case) | 25 (1.9%) | 10 (0.9%) | 15 (9.9%) | < 0.001 |
| Peripheral vascular complications (case) | 5 (0.4%) | 4 (0.3%) | 1 (0.7%) | 0.46 |
| Aortic complications (case) | 2 (0.2%) | 2 (0.2%) | 0 (0.0%) | 1.00 |
| False lumen thrombosis (case) | 6 (0.5%) | 5 (0.4%) | 1 (0.7%) | 0.52 |
| Respiratory complications (case) | 206 (15.6%) | 158 (13.5%) | 48 (31.8%) | < 0.001 |
| Postoperative bleeding (case) | 240 (18.2%) | 194 (16.6%) | 46 (30.5%) | < 0.001 |
| Total bleeding volume (mL) | 2635.0 (1632.5-3980.0) | 2475.0 (1600.0-3820.0) | 3250.0 (2000.0-5587.5) | 0.02 |
| Incision complications (case) | 34 (2.6%) | 28 (2.4%) | 6 (4.0%) | 0.27 |
| Deep infections (case) | 17 (1.3%) | 13 (1.1%) | 4 (2.6%) | 0.12 |
| Death (case) | 28 (2.1%) | 9 (0.8%) | 19 (12.6%) | < 0.001 |
| Abbreviations: ICU, Intensive Care Unit;  NOTE. The categorical variables in the table are represented by the number of cases (with percentage) and the continuous variables are expressed by the median (with the first quartile and the third quartile). P value was calculated using Mann-Whitney U test in continuous variables, and chi-square test or Fisher's exact test in categorical variables. | | | | |

| **Supplementary Table 2 Missing value details** | | |
| --- | --- | --- |
|  | Internal Validation Groups | External Validation Groups |
| Number of patients (case) | 1318 | 319 |
| LVEF | 115 (8.73%) | 21(6.58%) |
| Absolute value of leukocyte | 11 (0.83%) | 0 (0.00%) |
| Platelet | 13 (0.99%) | 0 (0.00%) |
| D-Dimmer | 89 (6.75%) | 8 (2.51%) |
| INR | 34 (2.58%) | 6 (1.88%) |
| APTT | 26 (1.97%) | 5 (1.57%) |
| ALT | 34 (2.58%) | 8 (2.51%) |
| Albumin | 66 (5.01%) | 8 (2.51%) |
| Fasting blood glucose | 102 (7.74%) | 2 (0.63%) |
| Abbreviations: LVEF, Left Ventricular Ejection Fraction; INR, International Normalized Ratio; APTT, Activated Partial Thromboplastin Time; ALT, Alanine Aminotransferase;  NOTE. The missing data is expressed in terms of the number of missing cases of the item and the proportion in the corresponding group. | | |

| **Supplementary Table 3 All Features Included in the Patient Data** | | | | |  |
| --- | --- | --- | --- | --- | --- |
| Gender, female (case) | | | | |  |
| Age (years) | |  | | |  |
| Information on admission | | | | |  |
|  | Body temperature (℃) | | | |  |
|  | Pulse (beats/min) | | | |  |
|  | Respiratory frequency (breaths/min) | | | |  |
|  | Height (cm) | | | |  |
|  | Weight (kg) | | | |  |
|  | Body mass index (kg/m^2^) | | | |  |
|  | Systolic pressure (mmHg) | | | |  |
|  | Diastolic pressure (mmHg) | | | |  |
|  | Cardiac arrest (case) | | | |  |
|  | Preoperative cardiopulmonary resuscitation (case) | | | |  |
| Symptoms |  | |  | | |
|  | With pain (case) | | | |  |
|  |  | | First pain in the chest (case) | | |
|  |  | | First pain in the chest and back (case) | | |
|  |  | | First pain in the back (case) | | |
|  |  | | First pain in the waist (case) | | |
|  |  | | First pain in the abdomen (case) | | |
|  |  | | First pain in the lower limb (case) | | |
|  |  | | Laceration pain (case) | | |
|  |  | | Acupuncture pain (case) | | |
|  |  | | Knife-like pain (case) | | |
|  | With symptoms involving carotid vessels (case) | | | |  |
|  | With symptoms involving coronary arteries (case) | | | |  |
|  | With symptoms involving spinal or lumbar artery (case) | | | |  |
|  | With symptoms involving renal artery (case) | | | |  |
|  | Oliguria (case) | | | |  |
|  | With symptoms involving superior mesenteric artery (case) | | | |  |
|  | With symptoms involving lower limb arteries (case) | | | |  |
|  | With other rare symptoms (case) | | | |  |
| Medical history | | | |  |  |
|  | Circulatory diseases history (case) | | | |  |
|  | Poor control of hypertension (case) | | | |  |
|  | Aortic trauma history (case) | | | |  |
|  | Valvular heart disease history (case) | | | |  |
|  | Congenital heart disease history (case) | | | |  |
|  | Pulmonary heart disease history (case) | | | |  |
|  | Pericardial effusion history (case) | | | |  |
|  | Hereditary diseases (case) | | | |  |
|  | Aortic valve malformation (case) | | | |  |
|  | Behçet’s disease (case) | | | |  |
|  | Shock history (case) | | | |  |
|  | Smoking history (case) | | | |  |
|  | Drinking history (case) | | | |  |
|  | Infectious diseases history (case) | | | |  |
|  | Allergy history (case) | | | |  |
|  | Family history (case) | | | |  |
|  | History of previous cardiac surgery (case) | | | |  |
|  | History of previous noncardiac surgery (case) | | | |  |
|  | Endocrine and metabolic diseases history (case) | | | |  |
|  | Nervous system diseases history (case) | | | |  |
|  | Peripheral vascular disease history (case) | | | |  |
|  | Digestive system diseases history (case) | | | |  |
|  | Urinary system diseases history (case) | | | |  |
|  | Hematopoietic diseases history (case) | | | |  |
|  | Muscle and joint diseases history (case) | | | |  |
|  | Immune system diseases history (case) | | | |  |
|  | Limb ischemia history (case) | | | |  |
| CTA results | |  | | |  |
|  | Aortic dissection (case) | | | |  |
|  | Aortic aneurysm (case) | | | |  |
|  | Intramural aortic hematoma (case) | | | |  |
|  | Aortic ulcer (case) | | | |  |
|  | Complex type (case) | | | |  |
|  | Left renal artery stenosis >50% (case) | | | |  |
|  | Right renal artery stenosis >50% (case) | | | |  |
| Echocardiographic results | | | | |  |
|  | Left ventricular end-systolic diameter (mm) | | | |  |
|  | Left ventricular end-diastolic diameter (mm) | | | |  |
|  | Ascending aortic diameter (mm) | | | |  |
|  | Aortic root diameter (mm) | | | |  |
|  | Left ventricular ejection fraction (%) | | | |  |
|  | Aortic dissection (case) | | | |  |
|  | Aortic valve stenosis (case) | | | |  |
|  | Aortic valve regurgitation (case) | | | |  |
|  | Pericardial tamponade (case) | | | |  |
| Electrocardiogram and X-ray results | | | | |  |
|  | Coronary artery involvement (case) | | | |  |
|  | Pleural effusion (case) | | | |  |
| Preoperative laboratory examination results | | | | |  |
|  | Absolute value of erythrocyte (10^12^/L) | | | |  |
|  | Absolute value of leukocyte (10^9^/L) | | | |  |
|  | Platelet (10^9^/L) | | | |  |
|  | Hemoglobin (g/L) | | | |  |
|  | High sensitivity troponin I (ng/mL) | | | |  |
|  | Myoglobin (ng/mL) | | | |  |
|  | CK-MB (ng/mL) | | | |  |
|  | Lactate dehydrogenase (U/L) | | | |  |
|  | INR | | | | |
|  | D-Dimmer (ng/mL) | | | |  |
|  | APTT (s) | | | |  |
|  | ACT (s) | | | |  |
|  | BNP (ng/mL) | | | |  |
|  | Arterial blood gas analysis results | | | |  |
|  |  | | pH | | |
|  |  | | PaO2 (mmHg) | | |
|  |  | | SaO2 (%) | | |
|  |  | | PaCO2 (mmHg) | | |
|  |  | | BE (mmol/L) | | |
|  |  | | Lactic acid (mmol/L) | | |
|  | Blood amylase (U/dL) | | | |  |
|  | Urine amylase (U/dL) | | | |  |
|  | ALT (U/mL) | | | |  |
|  | AST (U/mL) | | | |  |
|  | Albumin (g/mL) | | | |  |
|  | Creatinine (μmol/L) | | | |  |
|  | BUN (mmol/mL) | | | |  |
|  | eGFR (ml/min/1.73m^2^) | | | |  |
|  | Fasting blood glucose (mmol/L) | | | |  |
|  | Glycosylated hemoglobin (mmol/mL) | | | |  |
| Diagnosis |  | |  | | |
|  | Aortic dissection (case) | | | |  |
|  | Intramural aortic hematoma (case) | | | |  |
|  | Aortic ulcer (case) | | | |  |
|  | Aortic aneurysm (case) | | | |  |
|  |  | | Located above the thoracic aorta (case) | | |
|  |  | | Threatened rupture (case) | | |
|  |  | | Pseudoaneurysm (case) | | |
|  | Aortic occlusive disease (case) | | | |  |
|  | Aortic stenosis (case) | | | |  |
|  | Aorta coarctation (case) | | | |  |
|  | Congenital aortic disease (case) | | | |  |
|  | Aortic arch variation (case) | | | |  |
|  | Mitral valve insufficiency (case) | | | |  |
|  | Mitral valve stenosis (case) | | | |  |
|  | Aortic valve insufficiency (case) | | | |  |
|  | Aortic valve stenosis (case) | | | |  |
|  | Marfan syndrome (case) | | | |  |
|  | Hypertension (case) | | | |  |
|  | Coronary artery disease (case) | | | |  |
|  | Acute myocardial infarction (case) | | | |  |
|  | Congestive heart failure (case) | | | |  |
|  | Chronic respiratory disease (case) | | | |  |
|  | Diabetes (case) | | | |  |
|  | Renal cyst (case) | | | |  |
|  | Hepatic cyst (case) | | | |  |
|  | Cerebral infarction (case) | | | |  |
|  | Pericardial effusion (case) | | | |  |
|  | Pleural effusion (case) | | | |  |
|  | Sleep apnea (case) | | | |  |
| Surgery |  | |  | | |
|  | Emergency surgery (case) | | | |  |
